# Large-scale pharmacogenomics based drug discovery for ITGB3 dependent chemoresistance in mesenchymal lung cancer

**DOI:** 10.1186/s12943-018-0924-8

**Published:** 2018-12-18

**Authors:** Soon-Ki Hong, Haeseung Lee, Ok-Seon Kwon, Na-Young Song, Hyo-Ju Lee, Seungmin Kang, Jeong-Hwan Kim, Mirang Kim, Wankyu Kim, Hyuk-Jin Cha

**Affiliations:** 1 College of Pharmacy, Seoul National University, Seoul, 08826 Republic of Korea; 2 Department of Life Sciences, Ewha Womans University, Seoul, 03760 Republic of Korea; 3 College of Natural Sciences, Department of Life Sciences, Sogang University, Seoul, 04107 Republic of Korea; 4 Personalized Genomic Medicine Research Center, Korea Research Institute of Bioscience and Biotechnology, Daejeon, 34141 Republic of Korea

**Keywords:** Chemoresistance, Mesenchymal cancer, Pharmacogenomics, Drug repurposing, Biomarker, *ITGB3*, NF-κB, Atorvastatin, Systems pharmacology

## Abstract

Even when targets responsible for chemoresistance are identified, drug development is often hampered due to the poor druggability of these proteins. We systematically analyzed therapy-resistance with a large-scale cancer cell transcriptome and drug-response datasets and predicted the candidate drugs based on the gene expression profile. Our results implicated the epithelial–mesenchymal transition as a common mechanism underlying resistance to chemotherapeutic drugs. Notably, we identified *ITGB3*, whose expression was abundant in both drug resistance and mesenchymal status, as a promising target to overcome chemoresistance. We also confirmed that depletion of *ITGB3* sensitized cancer cells to conventional chemotherapeutic drugs by modulating the NF-κB signaling pathway. Considering the poor druggability of ITGB3 and the lack of feasible drugs to directly inhibit this protein, we took an in silico screening for drugs mimicking the transcriptome-level changes caused by knockdown of *ITGB3.* This approach successfully identified atorvastatin as a novel candidate for drug repurposing, paving an alternative path to drug screening that is applicable to undruggable targets.

**Electronic supplementary material:**

The online version of this article (10.1186/s12943-018-0924-8) contains supplementary material, which is available to authorized users.

## Main text

Recent studies in both in vitro cell and in vivo animal models demonstrated that the epithelial-mesenchymal transition (EMT), a major cause of metastasis, is closely associated with chemoresistance [1]. These are consistent with the reports that cancer patients with mesenchymal gene signatures have poor prognoses or exhibit therapy resistance [2]. However, due to the poor druggability of the EMT-associated proteins responsible for chemoresistance (e.g., ZEB1/2, SNAI2, SOX4, etc.), it is important to develop alternative strategies to make ‘undruggable but attractive targets’ druggable.

**Fig. 1:**
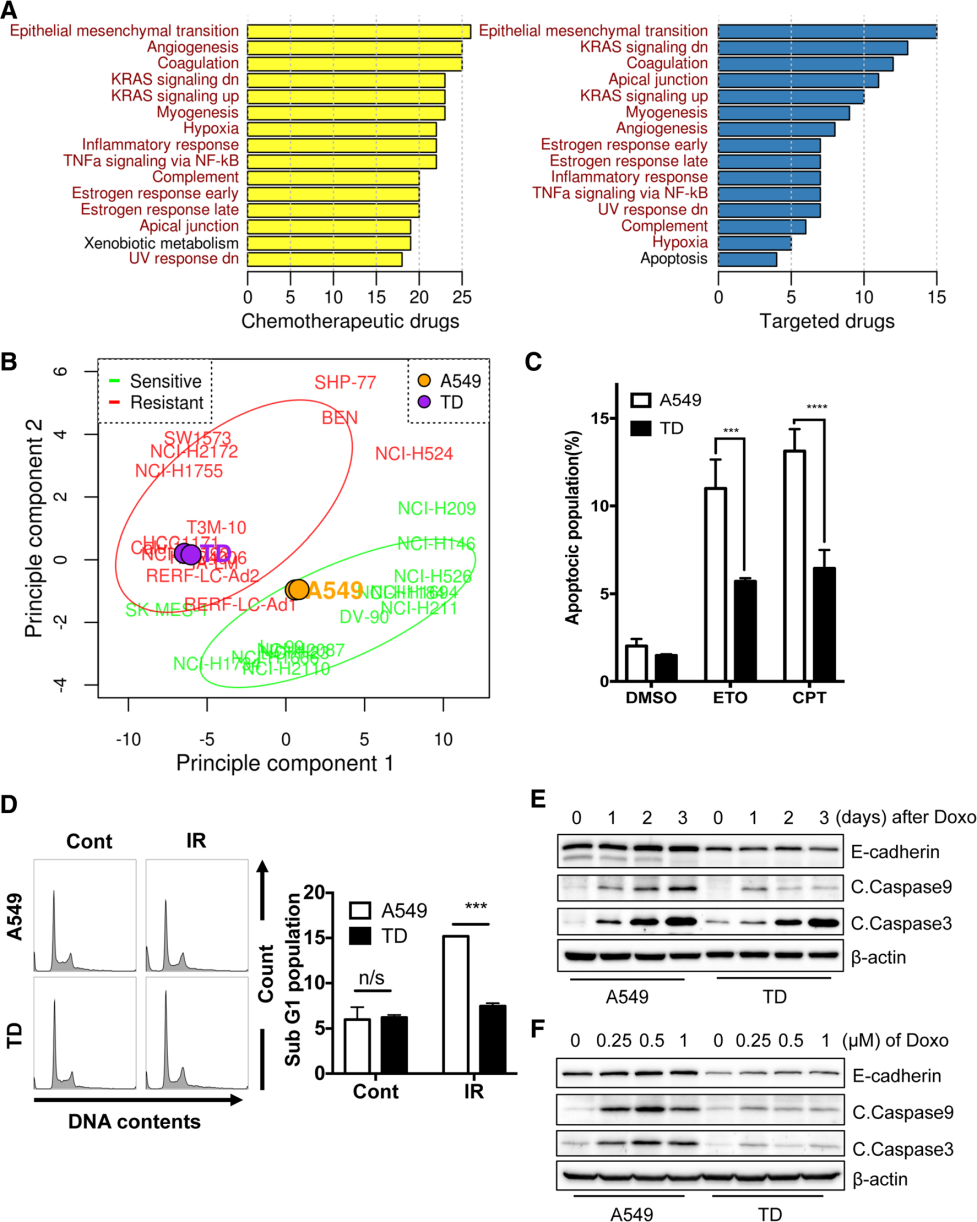
Epithelial mesenchymal transition as a common mechanism underlying anticancer drug resistance **a** Top 10 most up-regulated pathways in the resistance group across chemotherapeutic (left panel) and targeted drugs (right panel) are summarized as the number of drugs by which the corresponding pathway is significantly regulated. Significantly enriched pathways per a drug were selected through hypergeometric tests (FDR < 0.05) using the hallmark gene sets from MsigDB. **b** Clustering of A549 and TD cells together with other lung cancer cell lines from the resistant (red) and sensitive (green) groups for doxorubicin by Partial Least Square Discriminant Analysis (PLS-DA) based on known EMT-genes **c** Programed cell death was examined by Annexin V/7AAD staining after DMSO, etoposide (ETO: 80 μM) and Camptothecin (CPT: 1 μM) 48 h treatment. **d** Sub G1 population was measure by FACS at 48 h after IR. The quantified sub G1 population was presented as bar graph (right) **e** and **f** Immunoblotting for apoptosis marker such as cleaved caspase 3 and 9 (C.Caspase3 and 9) after doxorubicin (Doxo) treatment at indicative days (**e**) or concentration (**f**), β-actin and E-cadherin used for an equal loading control and epithelial marker

To elucidate the mechanisms underlying chemoresistance, we examined the gene expression profiles of 804 cancer cell lines, as well as their responses to anti-cancer drugs, using data from Cancer Therapeutics Response Portal (CTRP) (Additional file 1: Figure S1A). For each drug, cell lines were classified as resistant or sensitive group, and differentially expressed genes (DEGs) in each resistant group were selected (Additional file 1: Figure S1B). EMT was the most frequently up-regulated phathway in the resistant group across most chemotherapeutics (26 out of 32 drugs), and targeted drugs (15 out of 20 drugs) (Fig. 1a and Additional file 1: Figure S1C). Among the down-regulated genes, ‘immune & inflammatory response’ pathways were highly enriched (Additional file 1: Figure S1C). It would be noteworthy that upregulation of interferon signaling contributes to efficacy of chemotherapy [3]. Given the previous studies supporting EMT as a major chemoresistance mechanism [1], we chose to investigate potential targets among the EMT signature genes, of which the vast majority were up-regulated by chemotherapeutics (Additional file 1: Figure S1C). Using the mesenchymal-type lung cancer cells (A549TD; hereafter, TD) from the A549 lung cancer cell line [4] showing a clear gene signature of ‘hallmark of EMT’ (Additional file 1: Figure S1E) and high resistance to etoposide treatment [5] (Additional file 1: Figure S1F), we showed the expression profiles of TD cells cluster together with those of doxorubicin-resistant cell lines, whereas the parental line A549 with the sensitive cells (Fig. 1b). As predicted, high resistance of TD cells to other conventional chemoradiotherapies such as etoposide (ETO), camptothecin (CPT) (Fig. 1c), ionizing radiation (IR) (Fig. 1d) and doxorubicin (Fig. 1e-f), all of which trigger apoptosis by inducing DNA damage.

**Fig. 2:**
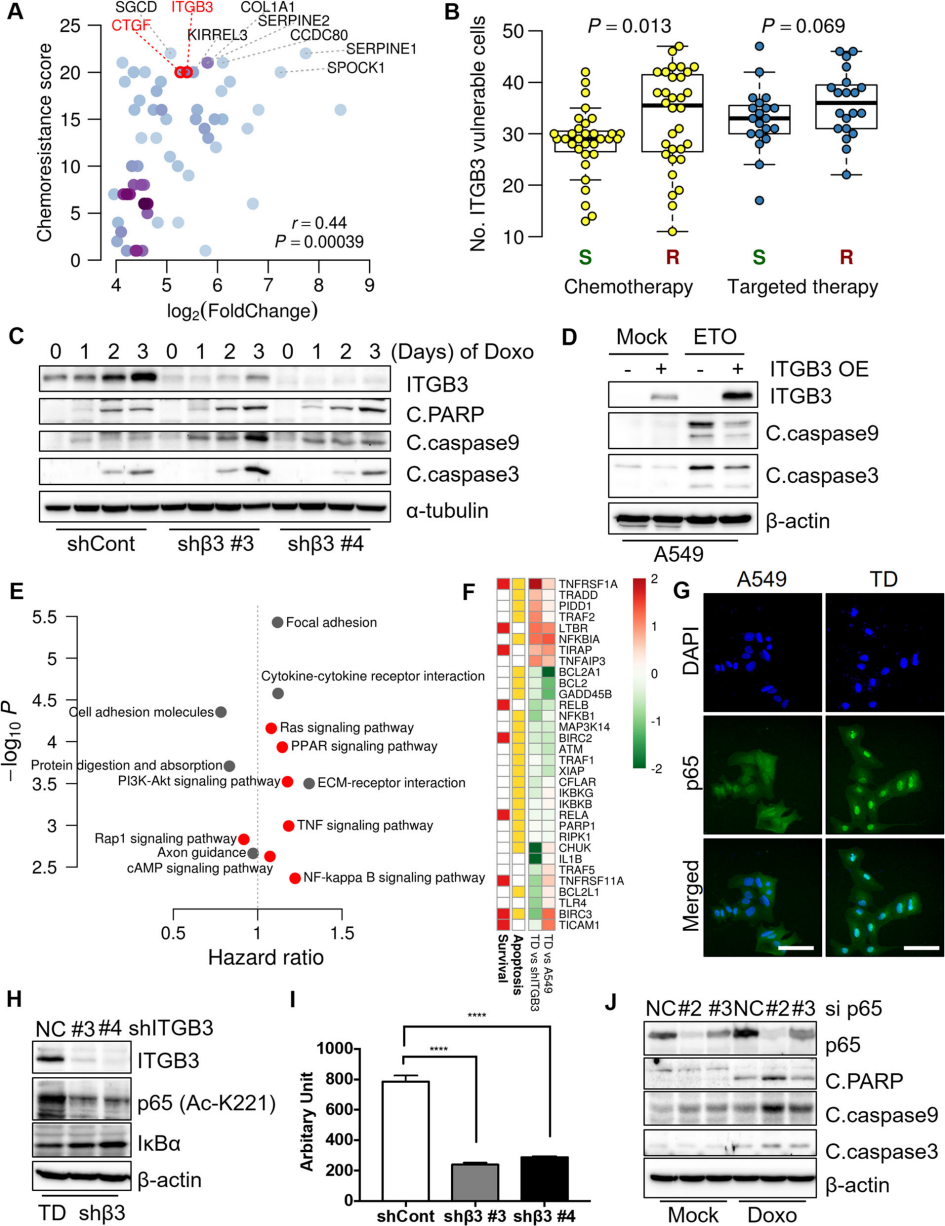
ITGB3-NFkB signaling contributes to acquisition of chemoresistance in mesenchymal lung cancer cell **a** The genes strongly associated with chemoresistance and increased expression in TD compared to A549 cell. Known EMT-genes are marked in red. **b** Distribution of the number of *ITGB3* vulnerable cells (dependency score < − 1) belonging to the sensitive (S) and resistant (R) group for 32 chemotherapeutic and 20 targeted drugs. (*P*-value by t-test). **c** Immunoblotting analysis for cleaved PARP (C.PARP), cleaved caspase 3 and 9 (C.Caspase3 and 9) of TD shCont, shβ #3 and shβ #4 after doxorubicin (Doxo) treatment at indicative days **d** Immunoblotting analysis for cleaved caspase 3 and 9 (C.Caspase3 and 9) after Etoposide (ETO, 80 μM) treatment with or without transient transfection of *ITGB3* in A549 **e** Enriched pathways (hypergeometric test, *q*-value < 0.1) and the median hazard ratio of the member genes in each pathway among the down-regulated genes by *ITGB3* depletion. Hazard ratio is calculated using TCGA LUAD patient dataset, and cancer signaling pathways are marked in red. **f** Expression change of NF-κB signaling genes (z-score, normalized log2 fold change) **g** Fluorescent microscopic images for p65 (Green) in A549 and TD cells. DAPI (Blue) for nuclear counterstaining, (The scale bars: 50 μm) **h** Immunoblotting analysis for IκB and acetylated p64 at lysine 221 (K221) in TD (shCont) and ITGB3 KD cells, β-actin for equal loading control **i** Luciferase reporter activity for NF-κB activity in TD (shCont) and ITGB3 KD cells (shβ#3 or shβ#4) **j** Immunoblotting analysis for p65, cleaved PARP, caspase 3 and 9 (C.PARP, C.Caspase3 and 9) after p65 knockdown with siRNAs (#2 or #3)

For prediction of genes for chemoresistance, we defined the frequency of a gene in the up-regulated DEGs as its ‘chemoresistance score’; this value was strongly correlated with the degree of overexpression in TD (Spearman correlation = 0.44, *P* < 0.0004, Fig. 2a). Two genes that satisfied all three of the criteria; i) genes frequently represented in the up DEGs of the 32 chemotherapeutic drugs; ii) genes overexpressed in TD cells; and iii) EMT-related genes were *ITGB3* and *CTGF* (Fig. 2a). Of note, the role of *CTGF* in chemoresistance and its antagonism for chemosensitization has been determined [6]. In particular, we found that the IC_50_ of doxorubicin is positively correlated with the *ITGB3* expression level (Additional file 1: Figure S2A). Analyzing a genome-scaling RNAi screening for 501 cancer cells, *Project Achilles* [7], 105 cells (21%) showed significant dependency on *ITGB3* (Additional file 1: Figure S2B), which was among the top ~ 10% vulnerable genes (Additional file 1: Figure S2C). Moreover, *ITGB3*-dependency was more significant in the resistant cells for most chemotherapeutic drugs (Fig. 2b and Additional file 1: Figure S2D). Such trend was most evident in eight chemotherapeutic drugs (Fisher’s method *P* < 1.1 × 10^− 5^, Additional file 1: Figure S2E). Consistently, loss of *ITGB3* in TD cells (shβ#3 and shβ#4) (Additional file 1: Figure S2F) increased sensitivity to doxorubicin treatment (Fig. 2c and Additional file 1: Figure S2G–I). Similar results were obtained with CPT, IR, and ETO (Additional file 1: Figure S2J-L). Given that expression of *ITGB3* was sufficient to restore the chemoresistance of shβ#3 TD cells (Additional file 1: Figure S2M) and even increase the chemoresistance of A549, the parental cell line of TD (Fig. 2d), we conclude that *ITGB3* expression is solely sufficient to induce chemoresistance.

Among the down-regulated pathways by *ITGB3* depletion, NF-κB was the signaling pathways most strongly associated with patients’ survival (Fig. 2e). Given that depletion of *ITGB3* down-regulated NF-κB-dependent survival factors (*IL8*, *XIAP*, *PLAU*, *BIRC2/3*, *BCL2*, or *BCL2L1*), and induced negative feedback regulators such as *NFKBIA* and *TNFAIP3* (encoding IκBα and A20 deubiquitinase, respectively), we hypothesized that inhibition of NF-κB signaling would be a key process required for cell sensitization (Fig. 2f and Additional file 1: Figure S3A). Consistently, highly chemoresistant TD cells exhibited higher levels of NF-κB activity than A549 cells, as determined by nuclear p65 localization (Fig. 2g and Additional file 1: Figure S3B), the protein level of IκBα (Additional file 1: Figure S3C) and NF-κB reporter activity (Additional file 1: Figure S3D). Furthermore, loss of *ITGB3* markedly attenuated NF-κB activity, as determined by NF-κB reporter activity (Fig. 2h) and acetylation of p65 (which is critical for its DNA-binding affinity) (Fig. 2i) as well as the level of nuclear p65 (Additional file 1: Figure S3E–F). In adverse, ectopic expression of *ITGB3* restored NF-κB activity, as determined by acetylation of p65 and level of IκB (Additional file 1: Figure S3G). Together, these data indicate that *ITGB3* expression is closely associated with NF-κB activity. According to this prediction that elevation of NF-κB activity by *ITGB3* expression could be a primary cause of the elevated hazard ratio (Fig. 2e), we assessed the cytotoxicity of doxorubicin following abrogation of NF-κB activity. Depletion of p65 with siRNA, was sufficient to sensitize cells to doxorubicin treatment (Fig. 2j), suggesting that the increase in NF-κB activity mediated by *ITGB3* expression is responsible for acquisition of chemoresistance.

**Fig. 3:**
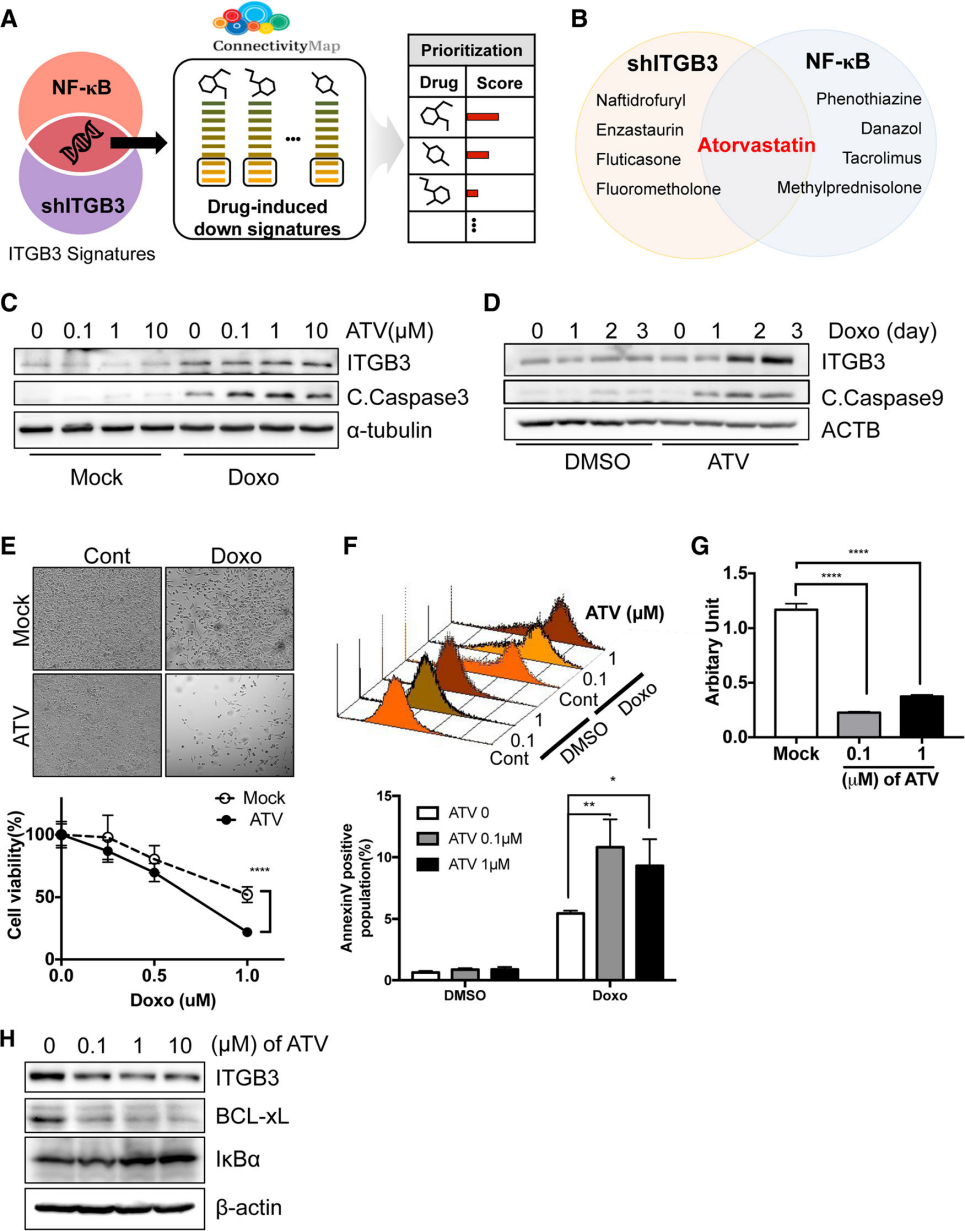
Atrovastatin sensitizes chemotherapy through modulating NF-κB **a** CMap approach to identify chemosensitizer drugs using two different signatures: i) down-regulated genes by ITGB3 depletion, and ii) the intersection of i) and NF-κB pathway genes **b** the candidate drug list predicted by the two signatures. Atorvastatin was commonly predicted by both signatures. **c** and **d** Immunoblotting analysis for cleaved caspase 3 or (C.Caspase3 or C.Caspase9) at indicative dose **c** of atorvastatin (ATV) or Days (**d**, with 0.1 μM of ATV) with doxorubicin (Doxo). α-tubulin or β-actin for equal loading control **e** Light microscopic images of TD cells with or without atorvastatin (ATV, 1 μM) after doxorubicin treatment (Doxo) (top), Graphical presentation of cell viability (bottom) **f** Flow cytometry for Annexin V staining at 24 h after indicative dose of Doxorubicin (Doxo) with 0.1 μM of ATV pretreatment (top), Graphical presentation of apoptotic cells (bottom) **g** Luciferase reporter activity for NF-κB activity in TD after indicative dose of atorvastatin (ATV) **h** Immunoblotting analysis for ITGB3, BCL-xL and IκB after indicative dose of atorvastatin (ATV) treatment in TD cells, β-actin for equal loading control

To predict chemosensitizing drug candidates, we leveraged drug-induced transcriptome data from the Connectivity Map (CMap) and searched for drugs with expression signatures similar to those of *ITGB3* depletion or NF-κB signaling inhibition (Fig. 3a). Among the candidate drugs, atorvastatin (ATV) was the only drug identified based on both *ITGB3* and NF-κB gene signatures (Fig. 3b and Additional file 1: Figure S4A-B). As predicted, pretreatment with ATV significantly sensitized TD cells to doxorubicin (Fig. 3c). It is noteworthy that, despite clear induction of *ITGB3* by treatment of doxorubicin, combined treatment of ATV and doxorubicin increased the rate of cell death (Fig. 3d), decreased cell viability (Fig. 3e) and apoptotic cell death (Fig. 3f). The increase in chemosensitivity following ATV treatment occurred in parallel with a reduction in NF-κB reporter activity (Fig. 3g). As similar as induction of *NFKBIA* (encoding IκBα) and suppression of *BCL2L1* (encoding BCL-xL) by *ITGB3* depletion (Additional file 1: Figure S3A), which may account for the decrease in NF-κB activity as well as pro-survival activity, short-course ATV treatment increased expression of IκBα and decreased the level of BCL-xL (Fig. 3h), suggesting that attenuation of the NF-κB–dependent pro-survival pathway by ATV leads to chemosensitization. Consistently, H460 cancer cells with mesenchymal gene expression (Additional file 1: Figure S5A) and high *ITGB3* and *IL6* expression (Additional file 1: Figure S5B), which was repressed by loss of *ITGB3* (Additional file 1: Figure S5C and D) became more chemosensitive by ATV treatment (Additional file 1: Figure S5F), lowing NF-κB activity (Additional file 1: Figure S5E). In other hand, H358 cancer cells with epithelial gene expression (Additional file 1: Figure S5G) were likely to acquire chemoresistance (Additional file 1: Figure S5H) and increased NF-κB activation (Additional file 1: Figure S5I) by *ITGB3* ectopic expression, which were weakened by ATV treatment (Additional file 1: Figure S5H and I). Conversely, depletion of *ITGB3* promoted chemosensitivity in H358 (Additional file 1: Figure S5J).

Most targets responsible for acquired chemoresistance in cancers, identified during extensive molecular mechanistic studies, remain undrugged [8] due to poor druggability or possible side effects by direct inhibition. Thus, we took advantage of CMap approach based on a large-scale drug-induced transcriptome dataset and identified ATV, one of the world’s best-selling drugs for hyperlipidemia, as a candidate drug for abrogating the pro-survival and chemoresistance effect of *ITGB3*; specifically, we showed that the transcriptional profile of ATV-treated cells was similar to that of *ITGB3* knockdown. Consistently, ATV has a radiosensitizing effect on prostate cancer cells [9]. Although the inhibitory effect of STATINs on NF-κB is varied markedly [10], our predictive analysis identified ATV as a top-ranking candidate, strongly validating our data-driven approach.

## Conclusions

By integrating pharmacogenomics and chemical genomic data, we successfully identified both a therapeutic target and a novel chemosensitizing drug to overcome resistance to multiple chemotherapeutic drugs. Our approach can be applied to a wide range of targets beyond those associated with EMT, paving an alternative path to drug discovery even for undruggable targets.

## Additional file

Additional file 1: Supplementary Materials, Methods, and Figures (Figure S1-S5). (PDF 5239 kb)

## Abbreviations

ATV: Atorvastatin
AUC: Area under fitted curve
CCLE: Cancer Cell Line Encyclopedia
CMap: Connectivity Map
CPT: Camptothecin
CS: Chemoresistance score
CTGF: Connective Tissue Growth Factor
CTRP: Cancer Therapeutics Response Portal
DEG: Differentially expressed gene
EMT: Epithelial–mesenchymal transition
GDC: Genomics Data Common
GDSC: Genomics of Drug Sensitivity in Cancer
ITGB3: Integrin beta 3
