# Supplementary material for: Large-scale pharmacogenomics based drug discovery for ITGB3 dependent chemoresistance in mesenchymal lung cancer
Source: Mol Cancer. 2018 Dec 18;17:175. doi: 10.1186/s12943-018-0924-8 (PMC6299529; doi:10.1186/s12943-018-0924-8)
Supplement: Supplementary file 1 — Supplementary Materials, Methods, and Figures (Figure S1-S5). (PDF 5239 kb) [file 12943_2018_924_MOESM1_ESM.pdf]

Supplemental information for

Large-scale pharmacogenomics approach to discover a novel chemosensitizer to abrogate the chemoresistance by ITGB3

Running title: Atorvastatin for ITGB3 dependent chemoresistance

Soon-Ki Hong, Haeseung Lee, Ok-Seon Kwon, Na-Young Song, Hyo-Ju Lee, Seungmin Kang, Jeong-Hwan Kim, Mirang Kim, Wankyu Kim, Hyuk-Jin Cha

This PDF file includes:

Supplemental Experimental Procedures

Supplemental Figures 1-5

Supplemental Experimental Procedures

Drug response and gene expression data of cancer cell lines

Drug response data of 886 cancer cell lines was downloaded from CTD^2 Data Portal (https://ocg.cancer.gov/programs/ctd2/data-portal). The cell viability values were converted into growth inhibition and adjusted to range in 0 ~ 100 % for fitting. Adjusted growth inhibition values were fitted by 4-parameter logistic regression method and low quality profiles (goodness of fitness < 0.7) were removed. To represent drug sensitivity as AUC (area under fitted curve), we selected the single range of concentrations that tested in the largest number of cells of each compound. AUC was normalized to range in 0 ~ 1 by maximum AUC that was assumed as 100 % growth inhibition in given range of concentrations. The basal-level gene expression profiles of cancer cell lines with drug response data were downloaded from Genomics Data Common (GDC, https://gdc.cancer.gov/) in the BAM file format. The read count was obtained using Subread package [21] with Gencode v19 annotation GTF file. Except the cell lines from non-human or ambiguous tissue types, 804 cancer cell lines' gene expression profiles were used for the analysis.

Drug resistance gene signature

In order to identify gene signature related to drug responses, cancer cell lines were divided into two groups according to their AUCs for each drug: the top 30% of the cell lines were classified as sensitive and the bottom 30% as resistant. For each drug, a list of highly expressed genes in the resistant group compared to the sensitive group was selected using DESeq2 [22] (fold change > 4 and FDR q-values < 0.01) and defined as the resistance signature.

Reagents and antibodies

siRNAs targeting *RELA(p65)* were obtained from Bioneer. shRNAs targeting *ITGB3* were obtained from Sigma Aldrich. Antibodies against E-cadherin (#4065), N-cadherin (#4061), cleaved caspase- 3(#9664), cleaved caspase-9(#9505), cleaved PARP (#5625) Phospho-Histone H2AX (Ser139) (#9718) and Integrin β3 (#4702) were obtained from Cell Signaling Technology. Antibodies against β-Actin (sc-47778), NF-kB subunit p65 (sc-372) and α-Tubulin (sc-8035) were obtained from Santa Cruz Biotechnology Inc. Antibody for BCL2 (551109) was obtained from BD Pharmingen™. NF-kB subunit p65 Acetyl-Lys221 (HW149) was bought from SAB.

Cell viability assay

To validate cell viability of cell after drug treatments, assays were carried out using the Enhanced Cell Viability Assay Kit EZ-CyTox (Daeil Lab Service Co., Ltd.) following manufacture's protocol. The absorbance of A450 and A650 of each well was measured using Epoch (BioTek).

Immunoblotting

Cells were lysed with RIPA buffer with 10uM sodium vanadate and 1mM protease inhibitor (Roche) and Immunoblotting were performed as described previously.

Real-time PCR

Total cellular RNA was extracted using Trizol (Invitrogen), followed by RT-PCR to generate the first strand cDNA, and the cDNA was subjected to SYBR Green-based Real-time PCR (Roche LightCycler 480 II).

FACS analysis

FACS caliber (BD Bioscience) was used for all the FACS analysis. For Annexin V staining, cells were washed twice with PBS and were stained with FITC-Annexin V and 7-AAD for 60 min at RT in dark. Cell death was analyzed with FACS according to apoptosis detection kit I protocol (BD Bioscience). For determining sub G1 population of cells after drug treatment, PI staining was performed. Cells were detached with 0.25 % trypsin and collected into micro centrifuge tube. Cells were washed by PBS twice and fixed by 70% EtOH during overnight. After that, PBS wash was done twice followed RNase 10mg/ml in PBS treatment. Samples were incubated in PI (1mg/ml) in PBS and analyzed by FACS in accordance with the manufacturer's instruction.

Nuclear extraction

To determine protein localization, cells were collected and washed by PBS. Cell pellet was lysed with buffer A (10 mM Hepes, 1.5mM $MgCl_2$, 10 mM KCl, 0.5 mM DTT with proteasome inhibitors) and incubated 10 min in ice. And solution was pumped with syringe (26G) 20 times and centrifuged at 13000 rpm for 1 min. supernatant was used for cytoplasmic fraction. The remained pellet was lysed by buffer C (20 mM Hepes, 25% Glycerol, 420 mM NaCl, 1.5mM $MgCl_2$, 0.5 mM EDTA, 0.5 mM DTT, 0.5 mM PMSF with proteasome inhibitor) and vortexed every 10min, 3times. The solution was centrifuged at 4 °C, 13000 rpm for 1min. supernatant was used for nuclear fraction sample.

Immunocytochemistry

Prepared cell were fixed by 4% formaldehyde and washed by TBS containing 0.1%

Triton X-100. Staining primary antibodies was progressed during overnight and secondary antibodies were incubated for 4 hours. Concentrations of Antibodies are determined according to the recommendation from suppliers. Finally, DAPI stained sample mounted on the slide.

Luciferase assay

To measure transcriptional activity of NF-κB, NF-κB binding elements were cloned into pGL3 vector (Promega). Cells were harvested at 48 hours after transfection and promoter reporter activity was determined using the Dual-Luciferase Assay System (Promega) following manufacture's protocol. Luciferase activity was measured by lumat BL9507 luminometer (Berthold technologies) and normalized to *Renilla* luciferase activity.

RNA sequencing and processing

We extracted Total RNA from the sample with Trizol following the manufacture instruction. Using TruSeq Stranded mRNA Library Prep Kit (Illumina, San Diego, CA), we made library construction. Shortly, the experiment proceeded as follows: (1) Strand cDNA synthesis (2) Strand synthesized using dUTPs instead of dTTPs (3) End repair, A-tailing and adaptor ligation (4) PCR amplification. (5) Each library was diluted to 8 pM for 76 cycles of paired-read sequencing (2 X 75bp) on the Illumina NextSeq 500 per the manufacturer's recommended protocol. Gene expression of lung cancer cell lines (A549, TD, and TD sh*ITGB3*) were processed and quantified as described above (CCLE RNA-sequencing pipeline) and the differentially expressed genes in each cell state were selected as a signature using DESeq2.

CMap approach

The CMap dataset[17] included an extensive catalog of 12,328 gene expression of 71 human cell lines in response to 20,413 chemical perturbations. The expressional change profiles (level 5, replicate-collapsed z-score matrix) and meta-data were downloaded from the Gene Expression Omnibus (accession id: GSE92742). The profile is a z-score vector that represents differential expression of all genes calculated by comparing the gene expression values of a single experiment condition (unique combination of compound, cell, treatment dose and time) with population control. From the total 205,034 profiles, we used only 12,588 profiles generated with more than three replicates, satisfying distil_cc_q75 ≥ 0.2 and pct_self_rank_q25 ≤ 5. We defined the 100 most down-regulated genes in each profile as a signature of the experiment. Depending on the number of experiments tested on a single compound, multiple signatures can be generated for the compound. To identify the drug candidates that mimic a particular phenotype such as knockdown of ITGB, we used the *jacard index* as a measure of signature similarity between phenotype and compound. After calculating the pairwise similarities, multiple similarity values of a compound are combined into a single score based on their rank distribution. The score is calculated as a negative logarithm of *P* value of hypergeometric test that quantifies the degree of over-representation of the compound's signatures within the top 10%.

Statistical analysis

The graphical data were presented as mean ± S.E.M. Statistical significance among the three groups and between groups was determined using analysis of variance (ANOVA) following Bonferroni post-test and Student's t-test respectively. The degree of

significance was indicated as $p < 0.05$ (*), $p < 0.01$ (**), $p < 0.001$ (***). Statistical analysis was performed with GraphPad Prism 7 software (https://www.graphpad.com/scientific-software/prism/).

Supplemental Figure legends

Figure. S1 (A) Number of cancer cells with drug response data by tissue type, and drug classes of the 52 anti-cancer agents used in this study. **(B)** Schematic overview of identifying gene signature for therapy-resistance. **(C)** Top 15 most and down-regulated pathways in the resistance group across chemotherapeutic (left panel) and targeted drugs (right panel) are summarized as the number of drugs by which the corresponding pathway is significantly regulated. Significantly enriched pathways per a drug were selected through hypergeometric tests (FDR <0.05) using the hallmark gene sets from MsigDB. **(D)** The expression changes (log fold change) of known EMT-associated genes in resistant cell group compared to sensitive cell group of each drug. **(E)** Differentially up-regulated genes in TD compared to A549 are associated to EMT signature. EMT hallmark gene set was obtained from MSigDB and enrichment plot was rendered by Gene Set Enrichment Analysis (GSEA). **(F)** Graphical presentation of cell viability after indicative dose of Etoposide (ETO) treatment in A549 and TD cells

Figure. S2 (A) Correlation between *ITGB3*'s gene expressions and doxorubicin's responses of each lung cancer cell line observed in the GDSC dataset **(B)** Histogram of DEMETER dependency scores of *ITGB3* for 501 cancer cell lines. It shows a negatively skewed distribution (D'Agostino test $P < 2.5 \times 10^{-3}$). **(C)** Distribution of the number of vulnerable cells (DEMETER score < -1) for each of all 17,098 genes in the *Project Achilles* dataset. **(D)** The distribution of the number of ITGB3 vulnerable cells across the resistant and sensitive cell groups in each of 32 chemotherapeutic and 20 targeted drugs. **(E)** Eight drugs showing significant difference of *ITGB3* dependency scores between their sensitive and resistant groups. 399 cancer cell lines commonly tested for

drug sensitivity and RNAi screen were used. The significance was expressed as the Student's t-test *P* value (shown in red). **(F)** mRNA expression level of *ITGB3* (left panel), light microscopic images (middle panels) and immunoblotting (right panel) of shCont, shβ#3 and shβ#4 **(G)** Light microscopic image of TD (shCont) and two *ITGB3* knockdown TD cells (shβ #3 and shβ #4) after doxorubicin 1μM (Doxo) at 72hours treatment **(H)** Graphical presentation of cell viability after indicated dose of Doxorubicin (Doxo) in TD (shCont) and *ITGB3* knockdown TD cells (shβ #3 and shβ #4) **(I)** Flow cytometry analysis for Annexin V of TD (shCont), shβ #3 and shβ #4 after 1μM of doxorubicin (Doxo) (left), Graphical presentation of Annexin V positive population (right) **(J-L)** Flow cytometry for Annexin V and 7-AAD **(J and L)** or sub G1 population **(K)** of TD (shCont), shβ #3 and shβ #4 after 1μM of camptothecin (CPT at 72 hours) (J), 10Gy of ionizing radiation (IR) (K) or 80μM of etoposide (ETO, at 72 hours) (left), Graphical presentation of cell death (right) **(M)** Immunoblotting for cleaved caspase 3 and 9 (C.Caspase3 and 9) after doxorubicin (Doxo) treatment in TD (shCont) or ITGB3 knockdown (shβ3#3) cells after 1μM of Doxorubicin (Doxo), β-actin for equal loading control

Figure. S3 (A) NF-kB signaling pathway that reflects changes (z-score) in gene expression due to *ITGB3* depletion in TD. Annotations on nodes and edges were obtained from KEGG and the network was rendered by cytoscape **(B)** Immunoblotting for p65, IκB and PARP from nuclear (Nuc) and cytoplasmic (Cyt) fraction of A549 (A) or TD (T) cells, PARP and β-actin used for nuclear and cytoplasmic loading control (**C)** Immunoblotting for ITGB3, p65 and IκB of A549 or TD cells, β-actin for equal protein loading control (**D)** Luciferase reporter activity for NF-κB activity of A549 or TD cells

(E) Fluorescent microscopic images for p65 (Green) in TD (shCont) and *ITGB3* knockdown TD cells (shβ #3 and shβ #4), DAPI (Blue) for nuclear counterstaining, (The scale bars: 50 μm) **(F)** Immunoblotting for ITGB3, p65, PARP and α-tubulin from nuclear (Nuc) and cytoplasmic (Cyt) fraction of TD cells (NC) and *ITGB3* knockdown TD cells (shβ #3 and shβ #4), PARP and α-tubulin used for nuclear and cytoplasmic loading control **(G)** Immunoblotting for ITGB3, acetylated p65 (Ac-K221) and IκB in TD cells (shCont) and *ITGB3* knockdown TD cells (shβ #3 and shβ #4) after ITGB3 ectopic expression (ITGB3 O.E), β-actin for equal protein loading

Figure. S4 Top 10 candidate drug list predicted by CMap approach based on the signature of *ITGB3* depletion **(A)** and NF-kB signaling signature **(B)**.

Figure. S5 (A) Relative mRNA expression of *CDH1* and *CDH2* in A549 and H460 cells **(B)** Relative mRNA expression of *ITGB3* and *IL6* in A549 and H460 cells **(C)** Luciferase reporter activity of NF-κB in H460 cells at 24 hours after introduction of ITGB3 siRNA **(D)** Relative mRNA expression of *ITGB3* and *IL6* in H460 cells 24 hours after introduction of ITGB3 siRNA **(E)** Luciferase reporter activity of NF-κB in H460 cells at 24 hours after 0.1μM ATV treatment **(F)** Immunoblotting analysis of H460 cells for apoptotic event markers (C.Caspase 3 and C.Caspase 9) after doxorubicin with or without atorvastatin (ATV, 0.1 μM) 12 hours pre-treatment, β-actin for equal protein loading **(G)** Relative mRNA expression of *CDH1, CDH2, Vimentin (Vim),* and *ITGB3* in A549, TD and H358 cells **(H)** Immunoblotting analysis of H358 cells (after ectopic expression of *ITGB3*) for C.Caspase 3 after doxorubicin with or without atorvastatin (ATV, 0.1 μM) 12 hours pre-treatment, β-actin for equal protein loading **(I)** NF-κB reporter activity in H358 cell at 48 hours after transient induction of

ITGB3 (left) and at 24 hours after 0.1μM ATV treatment (right) **(J)** Relative mRNA expression of *ITGB3* in A549, TD and H358 cells (left), Immunoblotting analysis for cleaved PARP (C.PARP) and cleaved caspase3 (C.Caspase3) in ITGB3 knockdown with siRNA (siITGB3) in H358 cell line after indicative dose of Doxorubicin (Doxo) (right), β-actin for equal protein loading

Figure S1

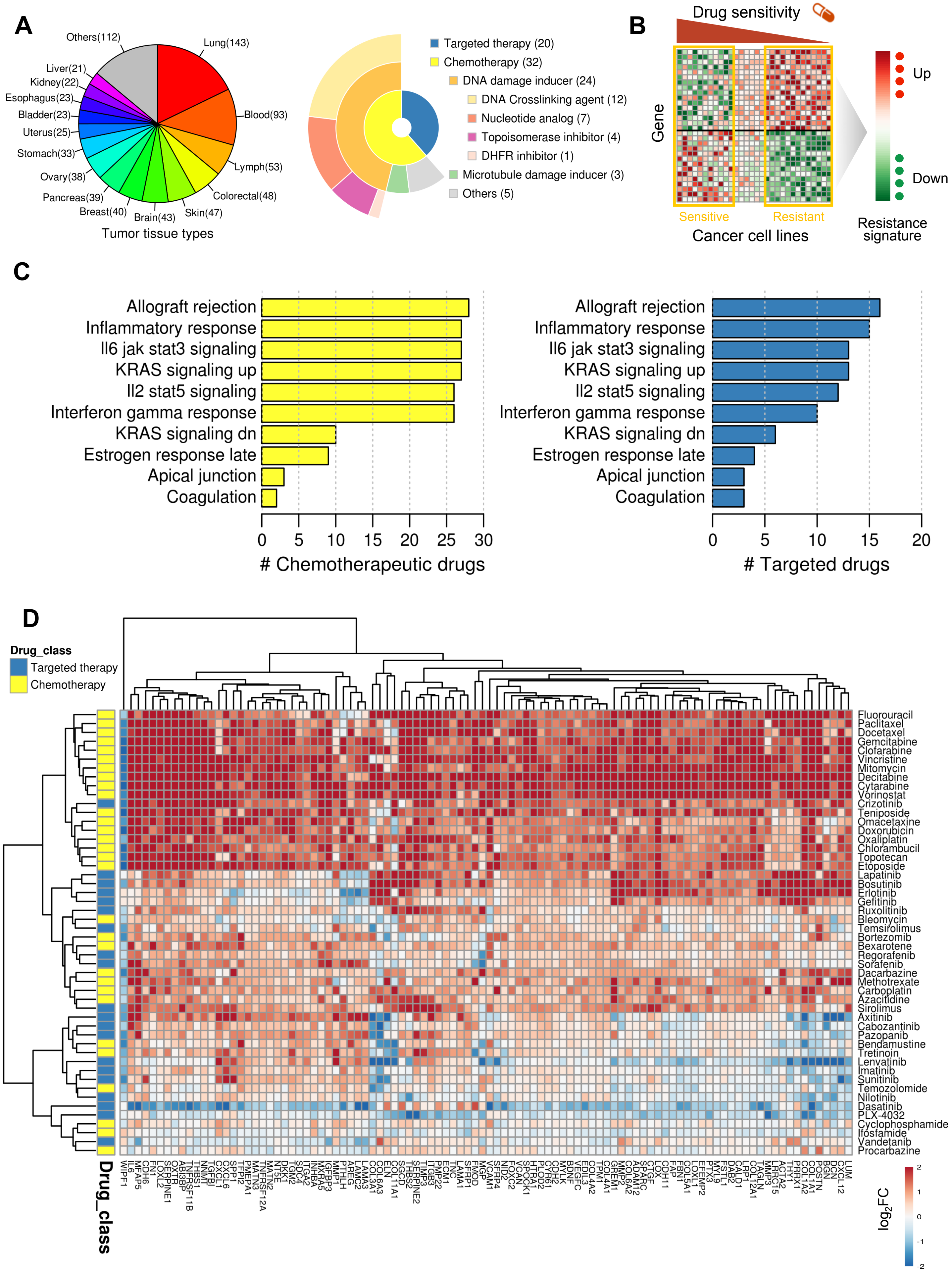
A
Others(112)
Lung(143)
Liver(21)
Kidney(22)
Esophagus(23)
Bladder(23)
Uterus(25)
Stomach(33)
Ovary(38)
Pancreas(39)
Breast(40)
Brain(43)
Skin(47)
Colorectal(48)
Lymph(53)
Blood(93)
Tumor tissue types
Targeted therapy (20)
Chemotherapy (32)
DNA damage inducer (24)
DNA Crosslinking agent (12)
Nucleotide analog (7)
Topoisomerase inhibitor (4)
DHFR inhibitor (1)
Microtubule damage inducer (3)
Others (5)
B
Drug sensitivity
Gene
Sensitive
Resistant
Cancer cell lines
Up
Down
Resistance signature
C
Allograft rejection
Inflammatory response
Il6 jak stat3 signaling
KRAS signaling up
Il2 stat5 signaling
Interferon gamma response
KRAS signaling dn
Estrogen response late
Apical junction
Coagulation
0 5 10 15 20 25 30
Chemotherapeutic drugs
0 5 10 15 20
Targeted drugs
D
Drug_class
Targeted therapy
Chemotherapy
Fluorouracil
Paclitaxel
Docetaxel
Gemcitabine
Clofarabine
Vincristine
Mitomycin
Decitabine
Cytarabine
Vorinostat
Crizotinib
Teniposide
Omacetaxine
Doxorubicin
Oxaliplatin
Chlorambucil
Topotecan
Etoposide
Lapatinib
Bosutinib
Erlotinib
Gefitinib
Ruxolitinib
Bleomycin
Temsirolimus
Bortezomib
Bexarotene
Regorafenib
Sorafenib
Dacarbazine
Methotrexate
Carboplatin
Azacitidine
Sirolimus
Axitinib
Cabozantinib
Pazopanib
Bendamustine
Tretinoin
Lenvatinib
Imatinib
Sunitinib
Temozolomide
Nilotinib
Dasatinib
PLX-4032
Cyclophosphamide
Ifosfamide
Vandetanib
Procarbazine
Drug_class
log2FC
2
1
0
-1
-2

E

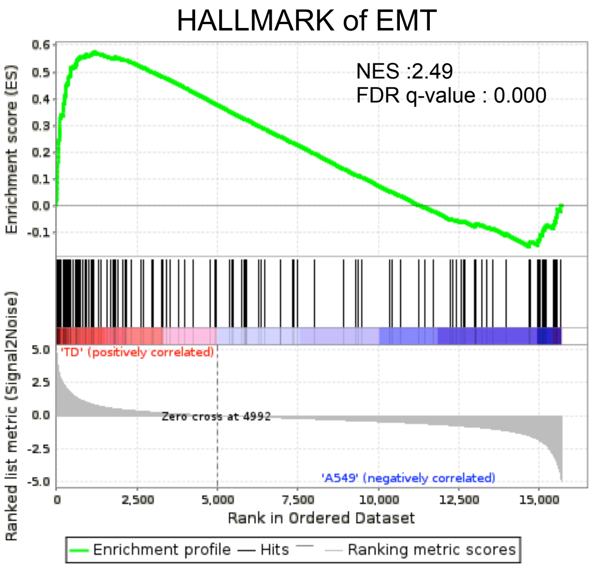
HALLMARK of EMT
NES :2.49
FDR q-value : 0.000
Enrichment score (ES)
Ranked list metric (Signal2Noise)
'TD' (positively correlated)
Zero cross at 4992
'A549' (negatively correlated)
Rank in Ordered Dataset
Enrichment profile — Hits — Ranking metric scores

F

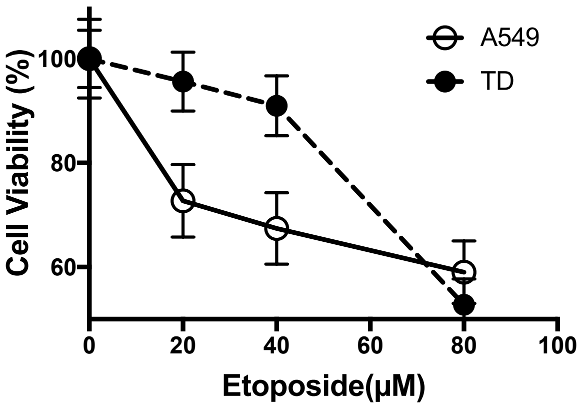
A549
TD
Cell Viability (%)
Etoposide(μM)

Figure S2

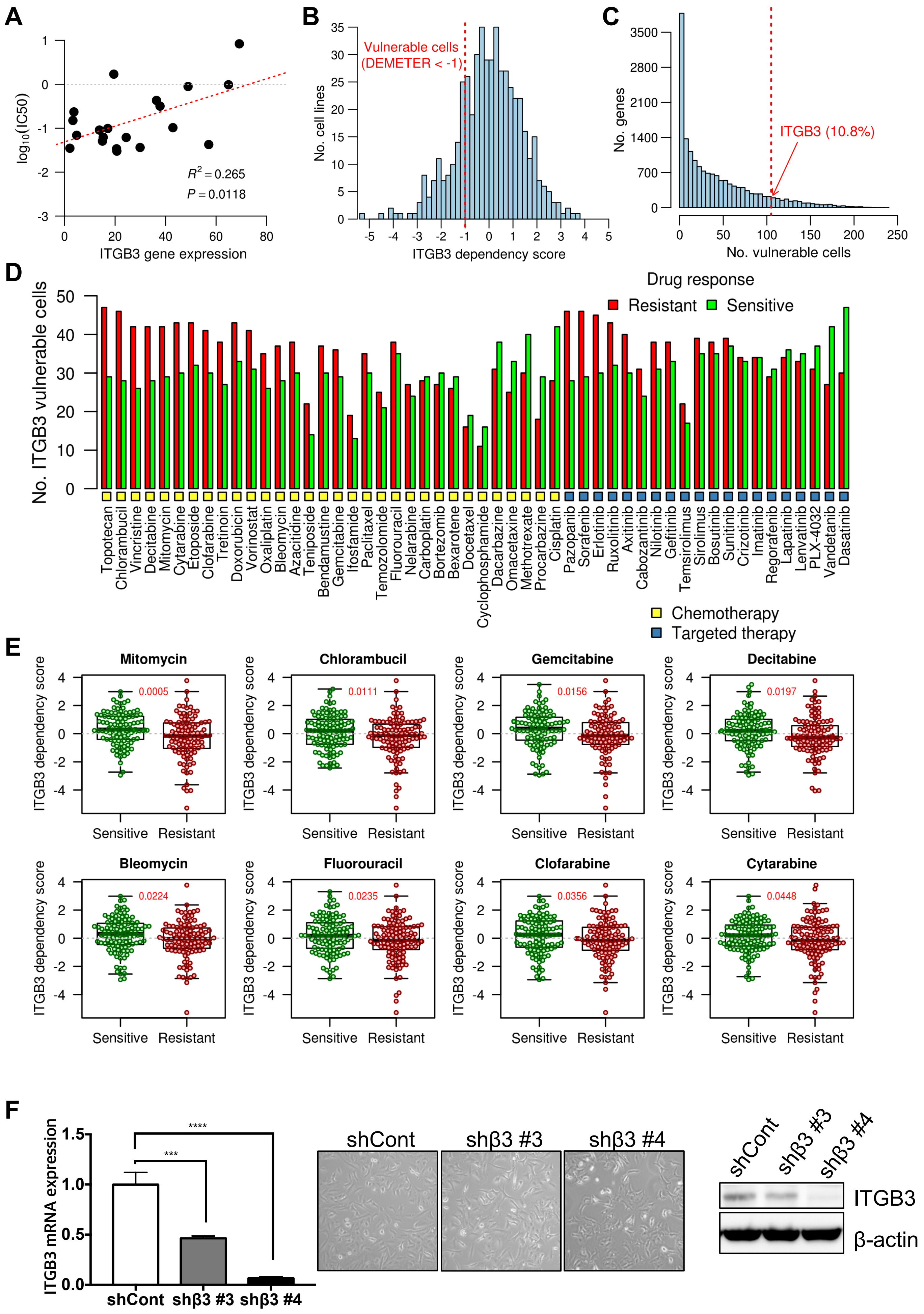

A
log10(IC50)
ITGB3 gene expression
R^2 = 0.265
P = 0.0118
B
Vulnerable cells
(DEMETER < -1)
No. cell lines
ITGB3 dependency score
C
No. genes
ITGB3 (10.8%)
No. vulnerable cells
D
No. ITGB3 vulnerable cells
Drug response
Resistant
Sensitive
Topotecan
Chlorambucil
Vincristine
Decitabine
Mitomycin
Cytarabine
Etoposide
Clofarabine
Tretinoin
Doxorubicin
Vorinostat
Oxaliplatin
Bleomycin
Azacitidine
Teniposide
Bendamustine
Gemcitabine
Ifosfamide
Paclitaxel
Temozolomide
Fluorouracil
Nelarabine
Carboplatin
Bortezomib
Bexarotene
Docetaxel
Cyclophosphamide
Dacarbazine
Omacetaxine
Methotrexate
Procarbazine
Cisplatin
Pazopanib
Sorafenib
Erlotinib
Ruxolitinib
Axitinib
Cabozantinib
Nilotinib
Gefitinib
Temsirolimus
Sirolimus
Bosutinib
Sunitinib
Crizotinib
Imatinib
Regorafenib
Lapatinib
Lenvatinib
PLX-4032
Vandetanib
Dasatinib
Chemotherapy
Targeted therapy
E
Mitomycin 0.0005
Chlorambucil 0.0111
Gemcitabine 0.0156
Decitabine 0.0197
Bleomycin 0.0224
Fluorouracil 0.0235
Clofarabine 0.0356
Cytarabine 0.0448
ITGB3 dependency score
Sensitive Resistant
F
ITGB3 mRNA expression
****
***
shCont shβ3 #3 shβ3 #4
ITGB3
β-actin

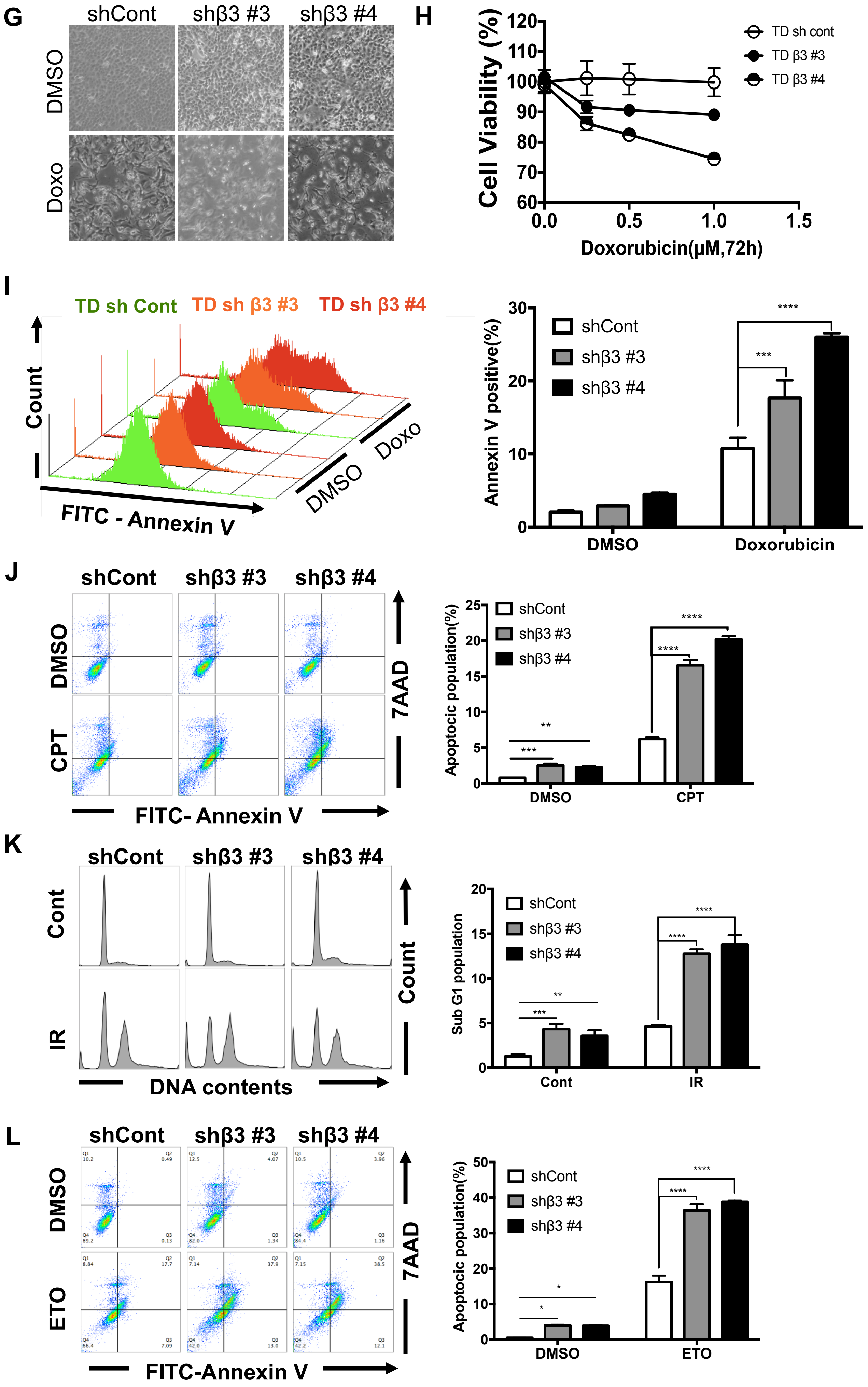
G
shCont
shβ3 #3
shβ3 #4
DMSO
Doxo
H
Cell Viability (%)
120
110
100
90
80
70
60
0.0
0.5
1.0
1.5
Doxorubicin(μM,72h)
TD sh cont
TD β3 #3
TD β3 #4
I
TD sh Cont
TD sh β3 #3
TD sh β3 #4
Count
FITC - Annexin V
DMSO
Doxo
Annexin V positive(%)
30
20
10
0
shCont
shβ3 #3
shβ3 #4
****
***
DMSO
Doxorubicin
J
shCont
shβ3 #3
shβ3 #4
DMSO
CPT
7AAD
FITC- Annexin V
Apoptocic population(%)
25
20
15
10
5
0
****
****
**
***
DMSO
CPT
K
shCont
shβ3 #3
shβ3 #4
Cont
IR
Count
DNA contents
Sub G1 population
20
15
10
5
0
****
****
**
***
Cont
IR
L
shCont
shβ3 #3
shβ3 #4
DMSO
ETO
7AAD
FITC-Annexin V
Apoptocic population(%)
50
40
30
20
10
0
****
****
*
*
DMSO
ETO

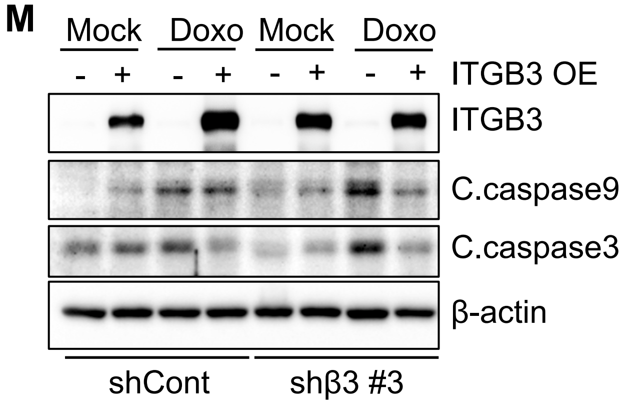
M
Mock
Doxo
Mock
Doxo
- + - + - + - +
ITGB3 OE
ITGB3
C.caspase9
C.caspase3
β-actin
shCont
shβ3 #3

Figure S3

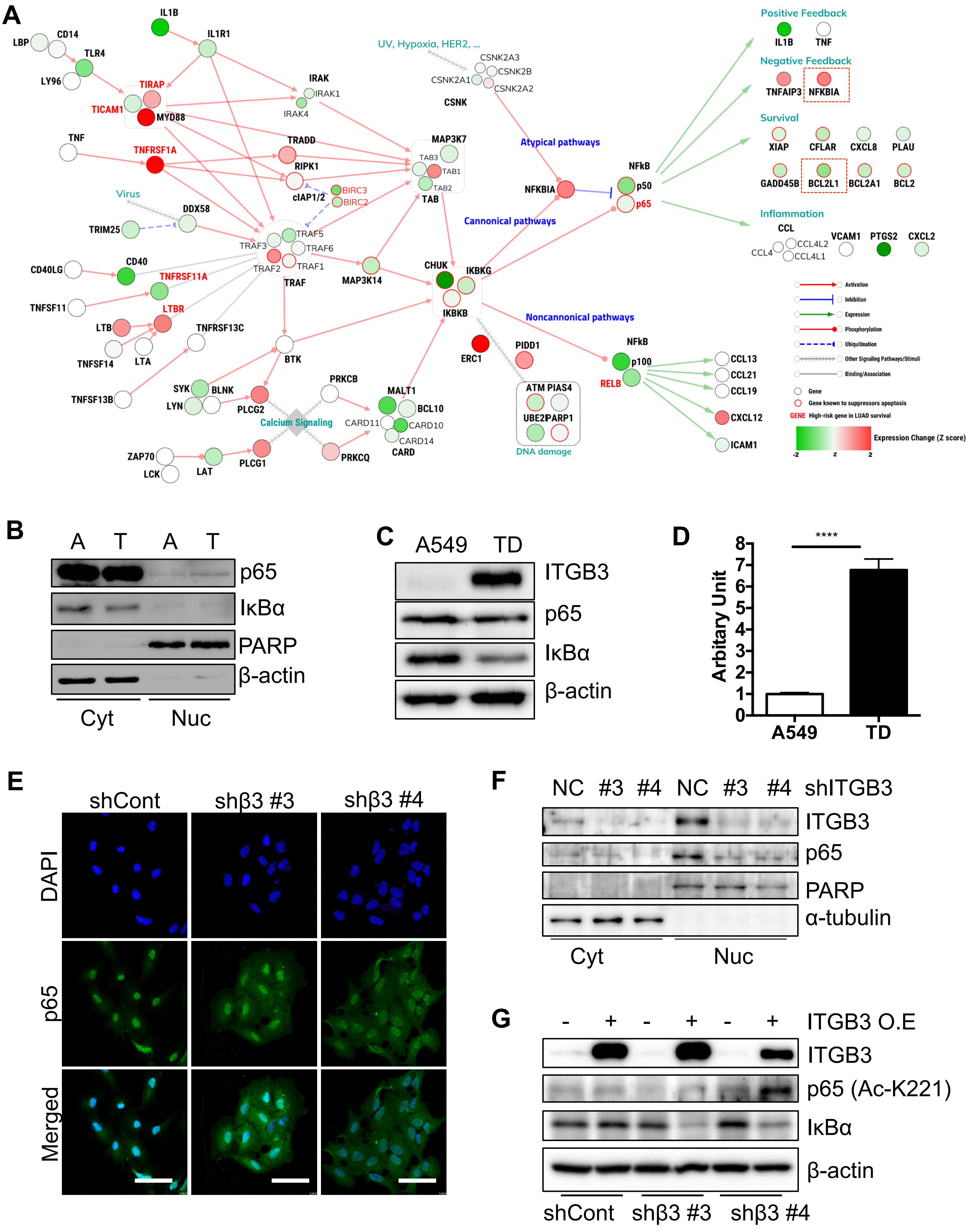
A
Positive Feedback
IL1B TNF
Negative Feedback
TNFAIP3 NFKBIA
Survival
XIAP CFLAR CXCL8 PLAU
GADD45B BCL2L1 BCL2A1 BCL2
Inflammation
CCL VCAM1 PTGS2 CXCL2
Atypical pathways
Cannonical pathways
Noncannonical pathways
Calcium Signaling
DNA damage
Virus
UV, Hypoxia, HER2, ...
Expression Change (Z score)
B
A T A T
p65
IκBα
PARP
β-actin
Cyt Nuc
C
A549 TD
ITGB3
p65
IκBα
β-actin
D
****
Arbitary Unit
A549 TD
E
shCont shβ3 #3 shβ3 #4
DAPI
p65
Merged
F
NC #3 #4 NC #3 #4 shITGB3
ITGB3
p65
PARP
α-tubulin
Cyt Nuc
G
- + - + - + ITGB3 O.E
ITGB3
p65 (Ac-K221)
IκBα
β-actin
shCont shβ3 #3 shβ3 #4

Figure S4

A

Rank	Name	Significant P
1	Enzastaurin	0.00087
2	Fluticasone	0.00125
3	Atorvastatin	0.00824
4	Fluorometholone	0.00998
5	Naftidrofuryl	0.00998
6	Forskolin	0.02185
7	Simvastatin	0.02185
8	Mestranol	0.02796
9	Mometasone	0.02796
10	Skf-81297	0.02796

B

Rank	Name	Significant P
1	Atorvastatin	0.00824
2	Danazol	0.00851
3	Tacrolimus	0.00851
4	Methylprednisolone	0.00998
5	Phenothiazine	0.00998
6	Triamcinolone-acetonide	0.00998
7	Dexamethasone	0.01253
8	Fluticasone-propionate	0.01577
9	Tamoxifen	0.01676
10	Vemurafenib	0.01787

Figure S5

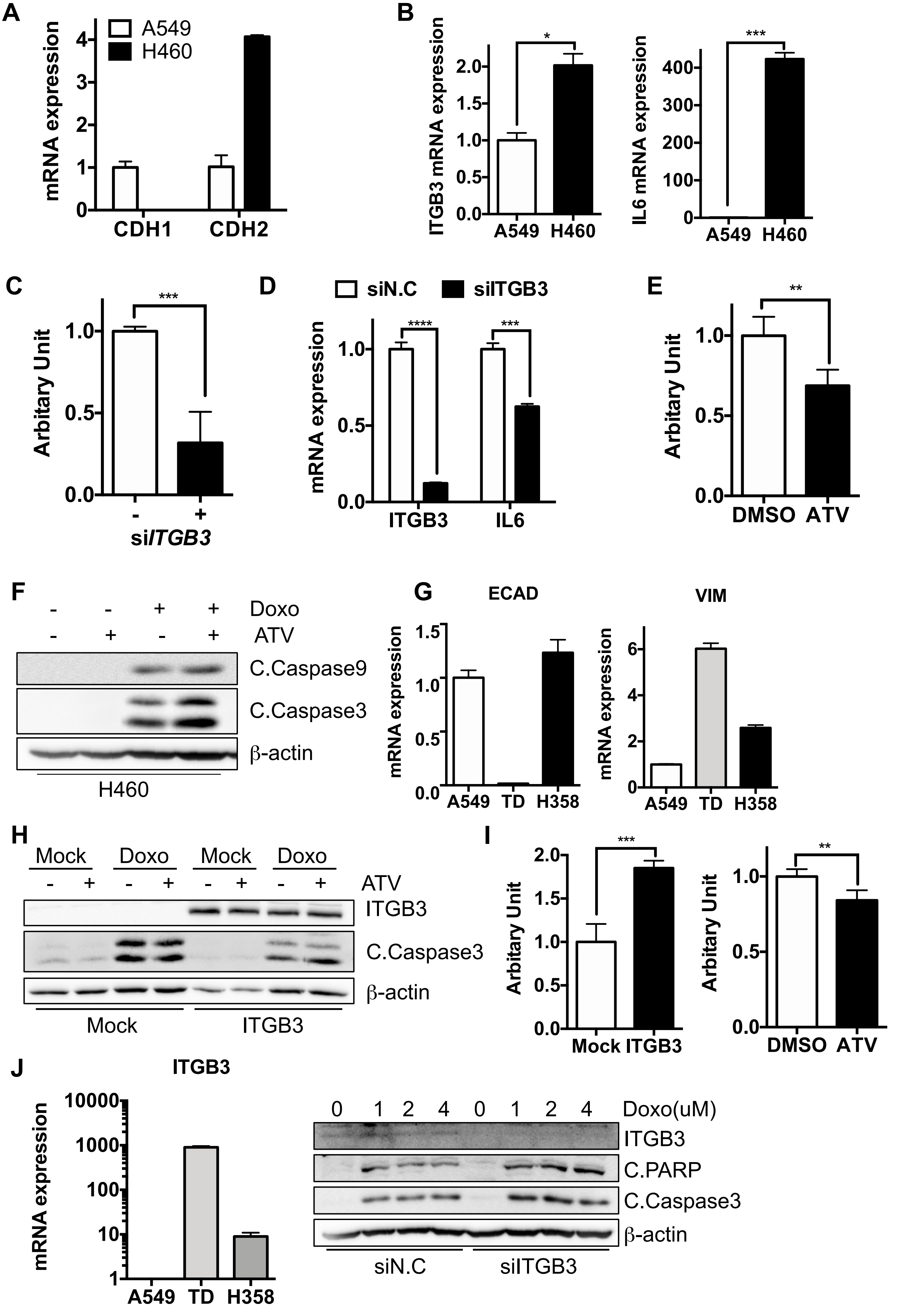
A
A549
H460
mRNA expression
CDH1
CDH2
B
ITGB3 mRNA expression
IL6 mRNA expression
A549
H460
C
Arbitary Unit
siITGB3
D
siN.C
siITGB3
mRNA expression
ITGB3
IL6
E
Arbitary Unit
DMSO
ATV
F
Doxo
ATV
C.Caspase9
C.Caspase3
β-actin
H460
G
ECAD
VIM
mRNA expression
A549
TD
H358
H
Mock
Doxo
ATV
ITGB3
C.Caspase3
β-actin
Mock
ITGB3
I
Arbitary Unit
Mock
ITGB3
DMSO
ATV
J
ITGB3
mRNA expression
A549
TD
H358
Doxo(uM)
ITGB3
C.PARP
C.Caspase3
β-actin
siN.C
siITGB3
